# Moving from control to elimination of schistosomiasis in sub-Saharan Africa: time to change and adapt strategies

**DOI:** 10.1186/s40249-017-0256-8

**Published:** 2017-02-20

**Authors:** Louis-Albert Tchuem Tchuenté, David Rollinson, J. Russell Stothard, David Molyneux

**Affiliations:** 1 National Programme for the Control of Schistosomiasis and STH, Ministry of Public Health, Yaoundé, Cameroon; 2 Centre for Schistosomiasis and Parasitology, University of Yaoundé I, Yaoundé, Cameroon; 3 Department of Life Sciences, The Natural History Museum, London, SW7 5BD UK; 4 Department of Parasitology, Liverpool School of Tropical Medicine, Pembroke Place, Liverpool, L3 5QA UK

**Keywords:** Schistosomiasis, Control, Elimination, Mapping, Diagnostics, Preventive chemotherapy, Mass drug administration, Sub-Saharan Africa

## Abstract

Schistosomiasis is a water borne parasitic disease of global importance and with ongoing control the disease endemic landscape is changing. In sub-Saharan Africa, for example, the landscape is becoming ever more heterogeneous as there are several species of *Schistosoma* that respond in different ways to ongoing preventive chemotherapy and the inter-sectoral interventions currently applied. The major focus of preventive chemotherapy is delivery of praziquantel by mass drug administration to those shown to be, or presumed to be, at-risk of infection and disease. In some countries, regional progress may be uneven but in certain locations there are very real prospects to transition from control into interruption of transmission, and ultimately elimination. To manage this transition requires reconsideration of some of the currently deployed diagnostic tools used in surveillance and downward realignment of existing prevalence thresholds to trigger mass treatment. A key challenge will be maintaining and if possible, expanding the current donation of praziquantel to currently overlooked groups, then judging when appropriate to move from mass drug administration to selective treatment. In so doing, this will ensure the health system is adapted, primed and shown to be cost-effective to respond to these changing disease dynamics as we move forward to 2020 targets and beyond.

**Electronic supplementary material:**

The online version of this article (doi:10.1186/s40249-017-0256-8) contains supplementary material, which is available to authorized users.

## Multilingual abstracts

Please see Additional file 1 for translations of the abstract into the six official working languages of the United States.

## Introduction

Schistosomiasis is a waterborne infection and is one of the most common parasitic diseases in the world, and is of public health global importance [1]. This disease has major health and socio-economic repercussions, and constitutes an important public health problem in developing countries as well as a significant hazard for visitors and travellers who visit disease endemic regions. Human schistosomiasis is caused by six species of schistosomes, i.e. *Schistosoma haematobium*, *S. mansoni*, *S. japonicum*, *S. mekongi*, *S. intercalatum* and *S. guineensis*; and is endemic in 78 countries [1, 2]. Of these six species, *S. haematobium* is responsible for urogenital schistosomiasis and has significant interactions with HIV and also HPV [3], whilst other species each cause intestinal or rectal schistosomiasis. It is estimated that 779 million people are at risk of infection, and about 250 million people are currently infected [2, 4]. The Global Burden of Disease study of 2010 attributed some 3.31 million disability-adjusted life years (DALYs) and 11 700 death per year to schistosomiasis, a mortality figure which has been challenged as a gross underestimate [5].

Schistosomiasis affects the poorest of the poor and infections are particularly abundant among people living in rural or deprived urban or peri-urban settings [6]. These populations typically have low socio-economic status with limited access to clean water and with inadequate sanitation provision [7, 8]. The morbidity caused by schistosomes is commonly associated with moderate-to-heavy egg-infection intensities and is progressive; as compared with any other age group, school-aged children and pre-school children are the most vulnerable groups to developing overt disease [9, 10]. These groups typically harbour the largest numbers of adult worms, with copious tissue entrapped eggs causing systematic and organ-specific inflammation, concomitantly when the consequences of this infection causes greatest physiological and developmental insult [2]. Studies have demonstrated that children can acquire schistosome infections within the first few months of life [11, 12], causing early life initial organ damage and altered development, mediated by fibrotic lesions around tissue-trapped eggs, manifesting overtly in adolescence and early adulthood [9, 13].

Successful schistosomiasis control programmes in Japan, China, Philippines, Brazil, Egypt and in some sub-Saharan African countries have shown that control of schistosomiasis with progression towards elimination of disease is feasible [14]. The recent impetus for schistosomiasis control has generated a greater political commitment, as well as an unprecedented opportunity for cost-effective action [15, 16]. This momentum has encouraged many countries to establish national action plans and programmes to control neglected tropical diseases (NTDs) [7, 17, 18].

Within the past decade, significant progress has been made on large scale treatments through integrated control of schistosomiasis and other NTDs, thanks to a number of international organizations, donor foundations, bilateral institutions and non-governmental organizations that responded to the WHO’s 2001 call for action [19]. Today, treatment with praziquantel [20] is cost-effective and ‘preventive chemotherapy’ is currently the strategy of choice and endorsed by WHO [9, 21]. With a support from the USAID and the UK Department for International Development (DFID), as well as the Bill and Melinda Gates Foundation, the pharmaceutical industry, and several not-for profit organizations, millions of children are regularly treated for schistosomiasis and other NTDs simultaneously, through coordinated use of anthelminthic drugs [22, 23].

In the past, a key bottleneck to implementation of preventive chemotherapy for control of schistosomiasis in sub-Saharan Africa was the limited access to praziquantel, either purchased or donated [24]. From 2002, with the expansion of activities of the Schistosomiasis Control Initiative, it was clear that the future need for large-scale quantities of praziquantel would grow [18]. In 2007, Merck KGaA pledged to donate 200 million tablets of praziquantel over 10 years through WHO. However, in 2012, Merck-KGaA committed to increase its donation to 250 million tablets of praziquantel per year until schistosomiasis is eliminated. To bolster this donation, additional amounts of praziquantel and resources for implementation were provided by other partners. Whilst there is now growing access to praziquantel for schistosomiasis control in sub-Saharan Africa, it is not at the level of projected requirements to reach all people at risk and requiring treatment [25]. Analysis of data reported on treatment coverage for schistosomiasis show that utilization of available praziquantel by NTD programmes is not yet optimal in many countries [22, 23]. Furthermore, special attention is needed to develop new access plans and reporting frameworks to vulnerable demographic groups in high-risk areas, particularly pre-school-aged children who are currently overlooked [10, 26].

However, the control of schistosomiasis is a long-term undertaking which involves several challenges. Current strategies were designed primarily for the control of morbidity due to schistosomiasis and were formulated over two decades ago when the availability of praziquantel was limited [27, 28]. The new impetus towards schistosomiasis elimination requires some modification, adaptation and even change of strategies [14, 29, 30], which concomitantly raises new challenges and points for consideration [31, 32]. This paper highlights the progress made and reviews the main challenges and requirements to move from control to elimination of schistosomiasis in sub-Saharan Africa.

## Schistosomiasis and the global health agenda

In 1975, the World Health Assembly (WHA) adopted the WHA 28.53 resolution calling for the preparation of guidelines and increased efforts in drug development, water projects and partner mobilization for schistosomiasis control [27]. The following year, in 1976, the WHA29.58 resolution urged endemic countries to consider the epidemiological aspects of schistosomiasis when planning and implementing water management schemes, and to undertake specific measures to prevent the spread of the disease into new areas and neighboring countries [33]. However, despite the existence of tools in the 1970s and 1980s, control of schistosomiasis was only sustained for a prolonged period in a few countries and almost no progress was made in sub-Saharan African countries, the most endemic part of the world. In the 1990s, interest in the control of schistosomiasis in Africa waned, and disease control was overshadowed by other health priorities [34] through an era of health sector reform and World Bank driven Structural Adjustment Programmes.

Recent years have witnessed an increased interest in the control/elimination of NTDs, and today the control of schistosomiasis has again become a priority on the agenda of many governments, donors, pharmaceutical companies and international agencies. This has been catalysed by several other WHA resolutions. In 2001, all member states of WHO endorsed the WHA54.19 resolution on schistosomiasis and STH, with the major objective of the regular treatment of at least 75% of all school-aged children at risk of morbidity by 2010 [28]. This resolution generated a greater political commitment and encouraged many countries to establish national action plans and programmes for schistosomiasis and STH control. Ten years later, in January 2012, WHO published an NTD Roadmap that set targets for the period 2012–2020, and described the strategic approach to accelerate work to overcome the global impact of NTDs. This roadmap identified preventive chemotherapy as a key strategy for tackling NTDs which responded to mass chemotherapy [21, 35]. At the same time (January 2012), partners and stakeholders (pharmaceutical companies, donors, endemic countries, Non-Governmental Organizations) endorsed the London declaration on NTDs, and committed to support the WHO roadmap and its 2020 targets for 10 NTDs. In response, the pharmaceutical sector committed to donate all required drugs for as long as necessary [36].

Still in 2012, WHO member states endorsed the WHA65.21 resolution on elimination of schistosomiasis, that called on all endemic countries to intensify control interventions and strengthen surveillance. Importantly, this resolution urged countries to embark on schistosomiasis elimination where possible [35]. This change of policy was a bold and important step towards the elimination of schistosomiasis. Finally, in 2013, the WHA66.12 resolution on NTDs urged countries to ensure continued country ownership of programmes for NTD prevention, control, elimination and eradication; to further strengthen disease surveillance system; to expand and implement appropriate interventions against NTDs; to advocate for funding; to strengthen capacity for NTD prevention, control, monitoring and evaluation; to devise plans for achieving and maintaining universal access to and coverage with interventions against NTDs, including provision of safe drinking-water, basic sanitation, health promotion and education [1].

Clean water provision, sanitation and hygiene (WASH) are critical components in the prevention and care for all NTDs scheduled for intensified control or elimination by 2020. For schistosomiasis, improved sanitation across the entire community to prevent contaminated faeces and urine from reaching surface water can reduce or eliminate transmission, by stopping worm eggs in faeces and urine from entering water–the snail habitat. Provision of safe water, sanitation and hygiene is one of the five key interventions within the global NTD roadmap. This requires a strong intersectoral collaboration. WASH providers must prioritize reduction of inequalities to align with the Sustainable Development Goals’ agenda, as developed in the recent WASH strategy to accelerate and sustain progress on NTDs [37].

The WHO NTD roadmap set three time-bound goals for the control or elimination of schistosomiasis. First, 2015 for the elimination of schistosomiasis in the Eastern Mediterranean Region, the Caribbean, Indonesia and the Mekong River basin. Second, 2020 for schistosomiasis elimination in the Americas and Western Pacific Regions; and potential elimination as a “public health problem” in multiple countries in Africa. Although schistosomiasis was not yet scheduled for elimination in sub-Saharan Africa by 2020, the roadmap envisaged potential elimination in selected countries or parts of countries where conditions were appropriate, such as Zanzibar (United Republic of Tanzania) where a concerted effort was on-going (SCORE project and China-Africa initiative). Finally, the roadmap set a potential global elimination of schistosomiasis as a “public health problem” by 2025 [1, 31, 36].

## Progress in the control of schistosomiasis in SSA

A key aspect within the WHO Roadmap was making the burden of schistosomiasis much more explicit which then allowed calculation and forecasting of future praziquantel requirements for each country. There has been substantial progress towards WHO Roadmap goals for schistosomiasis and regional targets, as the control of schistosomiasis has become a priority on the agenda of many governments. This momentum has encouraged many countries to establish national action plans and programmes to control NTDs [7, 17, 38]. By 2016, 36 African countries had developed and launched their national NTD master plans. With a support from USAID and UK Department of International Development (DFID) governments, as well as the Bill and Melinda Gates Foundation, the pharmaceutical industry, and many not-for profit organizations, the mapping of NTDs has been completed and millions of children are regularly treated for schistosomiasis and other NTDs.

### Mapping the schistosomiasis distribution

Although the African region bears a disproportionately high burden of schistosomiasis, the mapping of disease prevalence remained incomplete in many countries. In January 2014, the WHO Regional Office for Africa (AFRO) launched a mapping initiative targeting to the completion of the mapping of the five NTDs amenable to preventive chemotherapy (lymphatic filariasis, onchocerciasis, schistosomiasis, STH and trachoma) in all countries of the African region. Funded by the Bill and Melinda Gates Foundation, this project enabled the acceleration of the completion of Preventive Chemotherapy amenable NTD mapping in the WHO African region.

Through a coordinated NTD mapping framework, strong WHO AFRO support, the deployment of a pool of well-trained NTD expert and mapping specialists, and a strong commitment by governments, significant progress has been made in the mapping of PC NTDs in the African region within the past 3 years. By June 2016, mapping of PC NTDs, including schistosomiasis, was completed in 41 countries of the 47 countries of the WHO African region, and there remained only six countries where mapping was still ongoing: Algeria, Angola, Central Africa Republic, Ethiopia, South Africa, and South Sudan [39]. This achievement was a critical step to enable the commencement of interventions towards the 2020/2025 schistosomiasis control and elimination targets.

### Treatment

Considerable progress is being made in scaling-up preventive chemotherapy interventions in sub-Saharan Africa. With a support from USAID, DFID, BMGF, the pharmaceutical industry and many not for profit organizations, millions of children are regularly treated for schistosomiasis and other NTDs. Within the past 10 years, the number of people treated for schistosomiasis in the WHO African region has significantly increased from about 7 million in 2006 to more than 52 million in 2014, corresponding to a scaling up of coverage from 5.47% in 2006 to 20.13% in 2014 [23].

**Fig. 1:**
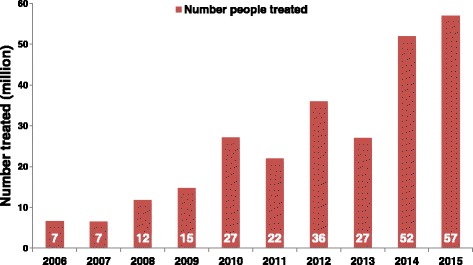
Evolution of the number of people treated for schistosomiasis and treatment coverage in the WHO African region, between 2006 and 2015

In the more recent report on schistosomiasis treatment worldwide, WHO estimated that the total number of people requiring treatment for schistosomiasis in 2015 was 218 700 000 (vs 258 875 452 in 2014), of whom 92.04% lived in the African Region. Reports on the annual progress on preventive chemotherapy interventions received in WHO by 20 September 2016 revealed that 27 African countries (vs 23 in 2014) had reported their treatment data for 2015 by then. From this interim report, the number of people treated in the Region was 57 400 000 in 2015 compared to 52 413 796 in 2014 and 26 489 501 in 2013. The number of school-age children that received treatment for schistosomiasis in 2015 was 46 600 000 (vs 43 725 454 in 2014), representing 81.2% of the total number of people treated in the African region [22, 23, 40]. Figure 1 illustrates the steady progress in schistosomiasis treatment in Africa since 2006. The increase of the number of treatments could be explained by several factors: the increased supply of praziquantel essentially from the Merck KGaA donation, new countries starting to implement preventive chemotherapy for schistosomiasis, geographical scale up of the treatment within countries and an improvement in the reporting.

The summary of the global update of preventive chemotherapy implementation in 2015 revealed a significant improvement in the treatment coverage, reaching for schistosomiasis up to 41.8 and 40.8% for school-aged children at the global and African regional levels, respectively. However, we are still very far from the target, and there is a need to strengthen programme performances to scale-up the preventive implementation and increase the coverage.

## Challenges and requirements

Moving from control to elimination of schistosomiasis is a paradigm shift that creates several challenges. Current interventions and strategies were designed for morbidity control or the elimination of schistosomiasis as a “public health problem” [9, 29]. The interruption of schistosomiasis transmission is a long-term undertaking but requires significant changes in the approach, design and strategies with a focus on reducing transmission and preventing reinfection. This involves several challenges such as implementation of intensified interventions, expansion of treatment coverage, use of alternative strategies, improvement of clean water supply, sanitation and hygiene, health education, funding for interventions, monitoring and evaluation, and strengthening of institutional capacities and surveillance response system. The present analysis highlights some of the key challenges and requirements for schistosomiasis elimination in sub-Saharan Africa.

### Moving from MDA focused intervention to complementary public-health interventions

The ultimate goal of all schistosomiasis intervention efforts should be the elimination of this infection. Several programmatic steps are recognized for the control and elimination of schistosomiasis [14]. These steps require specific interventions, including interventions for morbidity control and those for infection prevention. It is recommended that schistosomiasis endemic countries progressively scale-up their objective from control of morbidity to elimination as a public health problem, and finally interruption of transmission. While moving through these steps, activities should be reorganized gradually. Currently, morbidity control is the objective in many countries and interventions are limited to chemotherapy with praziquantel [23, 41]. However, it is known that treatment alone will not be sufficient to achieve the interruption of schistosomiasis transmission [42]. Therefore, if the elimination goals for schistosomiasis are to be met, endemic countries should adopt a final push approach combining intensified preventive chemotherapy and the implementation of complementary public-health, environmental and educational interventions. Such intensified preventive chemotherapy consists of implementing the distribution of praziquantel more frequently, and/or to extend the treatment to population groups that are different than those targeted so far [31, 32].

Complementary public-health interventions include health education for behaviour change, provision of safe water and sanitation, environmental management and snail control. This combined approach is recommended in areas approaching elimination as a public-health problem, and is essential when interruption of transmission is at the objective. In the Regional Strategic Plan for schistosomiasis, WHO/AFRO defined this approach as PHASE, standing for preventive chemotherapy, health education, access to clean water, sanitation improvement, and environmental snail control and focal mollusciciding [14]. Increasing access to safe water is an intervention that will significantly reduce the risk of schistosomiasis transmission. Its achievement requires inter-sectoral collaboration and partnership. However, most countries cannot raise the resources required to drastically increase safe water supply. Thus, in most schistosomiasis endemic countries, natural water bodies (many of which are infested with snails and infective schistosome cercariae, sometimes of zoonotic origin) continue to be the only sources of domestic water and high risk communities cannot avoid reinfections even if they were effectively treated. A further challenge is to address the needs of those where occupational exposure is a daily feature of tending, for example, to agricultural work and fishery [43].

Poor sanitation is a major contributor to transmission of schistosomiasis and causes rapid re-infection among treated children and adults. Improvement in waste disposal and a reduction in open defaecation is essential for achieving interruption of transmission. Improvement in sanitation not only contributes to prevention of transmission, but also to the prevention of many diarrhoeal diseases. Sensitization and mobilization of people to build and use latrines should be strengthened. There are two main strategies within WASH that feature participatory hygiene and sanitation transformation (PHAST) and community-led total sanitation (CLTS), however, neither of these approaches will effectively reduce the contamination of water sources by schistosome eggs in the urine. Environmental management for snail control has not been generally undertaken in the sub-Saharan African region due to cost limitations and lack of identification of the water bodies where this is feasible. As snail control is generally challenging especially in large water bodies, there is need to identify areas with high water contact and intensive schistosomiasis transmission so that targeted snail control can be limited to such locations. However, technical capacity and funding to implement reliable snail surveys is lacking in many countries. In China, new and effective snail control approaches, environmental modification (i.e. alteration of the ecological environments of the snails’ habitats to make their survival difficult) have been developed and adapted to the local situation in snail-infested areas. The current China-Africa cooperation for schistosomiasis elimination provides a platform to learn from Chinese experiences, in the control of intermediate snail hosts [44].

### Scaling up treatment

Although significant progress has been made over the recent years to regularly implement MDA in several countries, the global achievement is still distant from the WHO’s target of regular deworming of at least 75% of school-age children at risk. Indeed, it is estimated that the global coverage of schistosomiasis treatment in 2015 was only 28% [40]. In many countries, school-based deworming interventions still cover only a minority of children considered to be at risk despite the low cost of preventive chemotherapy and their significant impact on health. Despite the increase in drug donation, the major constraint to controlling schistosomiasis continues to be the limited access to praziquantel. In 2015, only nine countries have reached the target threshold treatment of at least 75% of school-age children in the African Region [40].

To reach the schistosomiasis elimination target, there is an urgent need to accelerate the extent of treatment to reach all individuals at risk. This extension of preventive treatment for schistosomiasis remains a serious challenge, and should be conducted at several levels. First, there is a need to accelerate the scaling up of mass drug administration to reach 100% geographical coverage and at least 75% of school-aged children in all endemic countries in the African region. This include a challenge to tackle the big countries such as Nigeria, Democratic Republic of Congo, Ethiopia and Tanzania which account for 60% of at risk population not yet entirely covered by preventive chemotherapy. Secondly, there is a need to extend treatment to the maximum number of out-of-school school-aged children. Children aged 5–14 years, who are the main target group of preventive chemotherapy, are relatively easily reached through school based deworming. However, most of out-of-school children are not reached through this platform. Special efforts should be made to extend treatment to this group. Thirdly, there is a need to extend preventive chemotherapy to adult populations. The available donated drugs are for school-aged children primarily, and therefore adults, especially high risk populations such as fishermen, irrigation workers, and women are not treated during deworming campaigns.

The risk factor of urogenital schistosomiasis for infection by HIV in women has been clearly demonstrated [45, 46], and adolescent girls and women therefore require treatment with praziquantel in areas endemic for *S. haematobium* more frequently than in non-endemic areas, to reduce the risk of development of genital lesions. Finally, it becomes urgent to recognize the importance of pre-school age children and their need for treatment. Although children aged less than 5 years can be already infected through passive water contacts sometimes at alarming levels [47, 48], they are currently not targeted by national chemotherapy campaigns because of a lack of suitable paediatric formulations of praziquantel [10, 26].

It is important to highlight that if the elimination goal is to be achieved for schistosomiasis, it will be essential to extend the preventive chemotherapy to all populations who need treatment, inclusive of pre-school aged children, school-aged children, as well as adults. Schistosomiasis does not just affect school-age children only, even though they may have the highest prevalence of infection, and possibly the heaviest disease burden. Without treatment of all those at risk or contributing to transmission it is not surprising that treatment limited to children has limited impact in this regard. Treating all the community will increase the impact of preventive chemotherapy, and will allow the reduction of schistosome reservoirs in the communities and accelerate the interruption of parasite transmission.

### Reaching hard to reach and vulnerable communities

Today, the coverage of the public-health interventions recommended by the World Health Organization against NTDs may be interpreted as a proxy for universal health coverage and shared prosperity. For schistosomiasis, universal health coverage means that all people in need should benefit from the preventive chemotherapy and other control/elimination interventions. The Sustainable Development Goals (SDGs) are reinforced by the commitment of global leaders to ensure that “no one is left behind” from development progress over the next 15 years. However, equity is not currently achieved for NTDs; hundreds million of the world’s most vulnerable, most disadvantaged people are still left behind, especially the poorest of the poor, who live in the remotest, hardest to reach parts of the countries or the world.

Hard to reach and vulnerable communities include communities that are poorly served by local health services, roads and transport facilities, itinerant fishing and nomadic communities, seasonal migrants, peri-urban settlers and those unwilling to accept health interventions (systematic non compliers). A good example are the challenges of reaching fishing communities along the large lakes such as Lake Albert, Lake Victoria and Lake Malawi that border several countries in Eastern Africa, including Uganda, Kenya, Tanzania and Malawi [49–51]. There are also areas inadequately covered with preventive chemotherapy due to civil unrest and conflict as well as health system crisis caused by recent Ebola outbreak in West Africa [52, 53].

### Adapting treatment to transmission dynamics: the need for alternative strategies

Schistosomes have a complex life cycle that requires a freshwater snail intermediate host and a vertebrate definitive host in which the parasites can undergo development. This ties transmission to landscapes where people and snails come together at the same water habitat. The success of the transmission depends on numerous factors, including biotic and abiotic features, such as climatic, physical and chemical factors that affect the survival and development of schistosome parasites and snail host populations [54] as well as socioeconomic and behavioural characteristics of the human community such as water contact behaviour and the adequacy of water and sanitation, which affect the frequency and intensity of exposure to infected water [55, 56]. The disease transmission is highly focal, and the endemicity varies significantly from one locality to another, and from one country to another. It is well known that the patterns and dynamics of transmission of schistosomiasis present tremendous complexity and variability between different foci and even within the same foci. The most significant determinants being water contact patterns, sanitation and hygiene levels, and the abundance and susceptibility of freshwater snail hosts.

**Fig. 2:**
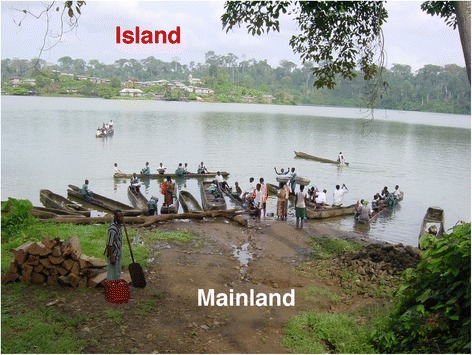
Intense water contact leading to high transmission dynamics of schistosomiasis in Barombi Kotto. Barombi Kotto, a village located in the South-West region of Cameroon, is divided in two parts; a mainland and an island. This photograph shows a view of the island from the shore of the mainland, and illustrates the intense water exposure of populations. There is no school in the island. All children leaving in the island go to school in the mainland. Therefore, they have contact with water at least twice per day, as they must cross the lake out and in. This frequent water exposure leads to rapid and high reinfections with schistosomiasis, that occur even from the same day of treatment in schools. Furthermore, there is no water supply in the island; the whole population relies on water from the lake, and 100% of people are at high risk of infections. The transmission dynamics and reinfection patterns are significantly different between populations from the island and those living on the mainland. Particular attention should be paid to such hotspots that require more regular and intensified interventions

The existence of schistosome hotspots–i.e. transmission areas where prevalence and intensities remain high despite repeated rounds of mass drug administration–has been demonstrated in several countries [57, 58]. For example, a number of villages near Lake Albert have shown to maintain very high levels of infection with *S. mansoni* following several years of chemotherapy with praziquantel [59]. Similar observations of hotspots infections despite repeated treatments have been reported in several other countries such as Tanzania/Zanzibar, Mali, Kenya, and Cameroon. In Cameroon, we observed hotspots of transmission in several localities around lakes and dams such as Barombi Kotto in the South West region and Malantouen in the West region, where water contacts are highly intense and lead to high reinfection patterns. In these foci the prevalence rapidly returns near to the initial level within 6–12 months post-treatment (Fig. 2).

To be efficient, preventive chemotherapy should be repeated more frequently in such hotspots, at least twice per year. However, the current recommended treatment strategy does not consider the diversity of transmission dynamics, reinfection patterns and the special features of schistosomiasis transmission foci. With the shift towards schistosomiasis elimination, there is a need to adapt treatment strategies to the different types of transmission settings. Urban schistosomiasis may also require more intense and frequent interventions.

### Mapping quality and uncertainties

The mapping of NTDs is a critical step in understanding where at-risk populations live in order to target effectively available resources and to achieve maximum impact on disease burden [60]. Without reliable mapping information, countries are not able to plan interventions. Accurate mapping of disease distribution is therefore a prerequisite for effective implementation of interventions to reduce the burden of schistosomiasis [57, 58, 61]. Within the past 10 years, significant progress has been made in the mapping of schistosomiasis in the African region, and mapping was completed in about 40 countries. This exercise was supported by various organizations, funders, partners, and research institutions in different countries.

In the mapping design for schistosomiasis, the health district is the implementation unit, and a subsample of up to five schools are generally selected for the surveys. Due to the high focality of schistosomiasis transmission, sub-districts may be considered in certain circumstances. However, financial resources being a major constraint, not every sub-district in a district can be mapped independently. The mapping design may combine several sub districts into mapping units, where transmission is likely to be similar, according to ecological factors affecting schistosomiasis transmission. This may lead to some uncertainties if the site selection and sample size are not properly undertaken. Indeed, selection of schools should be purposive and should be guided by previous knowledge in the areas where transmission is known, suspected or more likely to occur, such as proximity to lakes, streams, and water bodies. Schools should not be selected in the same locality, but selection should consider geographical distribution of schools in order to be representative within the health district. Importantly, due to the high focality of schistosomiasis transmission, random selection of schools should be avoided. However, different approaches were used in some countries where national surveys were conducted using random selection of schools in health districts, or using remote sensing technologies to predict schistosomiasis distribution. Studies have demonstrated that using predictive mapping alone does not provide reliable information for mass drug administration planning, resulting in overtreatment in some areas and most importantly under-treatment in areas that needed it most [62]. This raises concerns about the accuracy of various mapping data resulting from less robust techniques that have been used in several countries.

Because of the highly focal distribution of the disease, there is a need for more accurate mapping to deepen the understanding of the distribution of schistosomiasis and snails in the country, which should guide programme decision making for mass drug administration. Furthermore, the maps should be dynamic entities that change with time as control progresses, necessitating refinement of tools for updating the original disease maps. As elimination moves forward there will likely be a need to map more geographic points, with an optimum to get to all schools within health unit. Currently mapping level ratio of surveyed to non-surveyed schools if about 1:10, but recent work in Namibia using rapid diagnostic tests have decreased to 1:4, so there is quite a bit more surveillance could be needed when we are looking for the possibility of any evidence of having schistosomiasis [57]. Ideally, a knowledge of water contact sites and an understanding of local transmission should guide mapping decisions and interventions [63].

### Redefining disease endemicity and focality for eligibility for MDA

The eligibility of health districts for MDA implementation is determined by the disease endemicity levels which are generally estimated by the disease prevalence [9]. For each implementation or mapping unit, one prevalence will be estimated and the entire district will be classified as non-endemic, low, moderate or high-risk area. The treatment strategy will be decided based on this classification. For schistosomiasis, the initial mapping is done by collecting stool and/or urine samples in about five schools per district [64]. The disease prevalence of the districts is calculated as the mean prevalence of all samples from each district. This district will then be classified according to the level of this mean prevalence. Currently, schistosomiasis endemicity maps are produced on this basis, as well as the subsequent decision to implement preventive chemotherapy or not.

Although this WHO recommended method to estimate district endemicity may have been suitable in the past, within the context of morbidity control and paucity of drug availability and funding, it may not be suitable now elimination is the goal. Indeed, due to the high focality of schistosomiasis transmission, there may exist significant difference in infection prevalence between schools within the same districts. With such a mixture of low and high prevalence, considering only the mean prevalence may lead to an underestimation of the disease occurrence within some districts, resulting to their exclusion for treatment. For example, a district with one school having 49% prevalence and four schools exhibiting 0% prevalence each, will have a mean prevalence of 9.8%. As this mean prevalence is below 10%, this district will be classified on the map as not eligible for mass drug administration. The consequence would be that in parts of this districts populations will suffer for schistosomiasis and its morbidity without intervention from the national authorities. It is therefore necessary for national programmes to have the detailed distribution of the disease at the various sub-districts and schools’ levels to guide treatment decisions and avoid misclassifications [57, 63].

To assess the impact of the current determination of endemicity level on treatment decisions, we conducted a detailed analysis of the recent mapping data in Cameroon, comparing the estimation of district endemicity levels using the mean prevalence in one hand, and the maximum school prevalence for schistosomiasis per health district on the other. The results showed that over the total 189 districts mapped, 47 (24.9%) changed their endemicity classification when considering either the mean school prevalence or the higher school prevalence. Detailed analysis of these 47 districts revealed that when considering the mean prevalence, 44.7% of the districts (*n* = 21) had an overall prevalence of <10%, and should therefore be entirely excluded for mass drug administration, despite the fact that in some localities within these districts there exist high transmission foci, with a school prevalence up to 52.8%.

For the remaining 26 districts of the 57, they ranged all within the moderate-risk community group when using the mean prevalence estimates. However, when using the higher school prevalence, almost all districts except one (96.2%) changed category, moving from moderate-risk to high-risk communities.

**Fig. 3:**
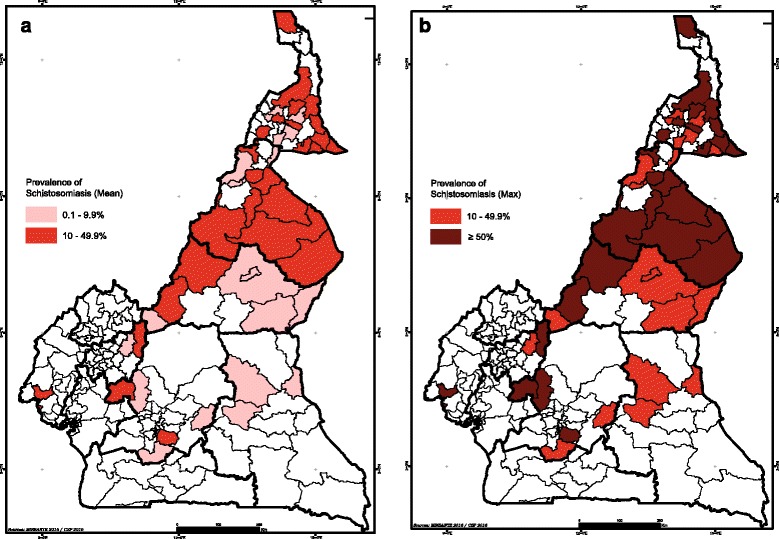
Comparison of district endemicity level/classification using either the mean prevalence of schistosomiasis per district (**a**) or the higher school prevalence within the district (**b**) in Cameroon

**Fig. 4:**
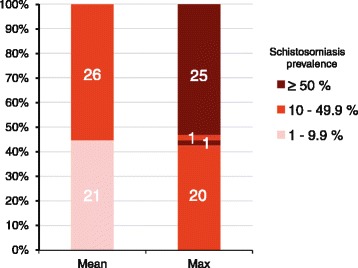
The changing of health district endemicity category for schistosomiasis in Cameroon, from lower-risk (*rose bars*) to moderate-risk (*red bars*) and high-risk (*dark red bars*), when moving from using the current recommended mean district prevalence (Mean) to using the maximum school prevalence within the district (Max). The number of districts per category are reported in the corresponding bars

These results, illustrated in Figs. 3 and 4, suggested that the current method of estimation of district endemicity significantly underestimates the disease transmission levels, and therefore reduced the treatment interventions. This underestimation and its impact on the programme policy decision showed that the way of determining district endemicity and eligibility to MDA is not suitable in a context of schistosomiasis elimination goal, calling for a reassessment of the current policy.

### The need to change (the current) treatment thresholds

Because morbidity is typically associated with increasing worm burden (and entrapment of eggs) rather than the absence or presence of infection, prevalence is commonly combined with worm burden (intensity of infection) to assess the epidemiological situation for schistosomiasis. Worm burden is commonly measured by the number of eggs per gram (EPG) of faeces or eggs per 10 ml of urine [13]. Prevalence and intensity of infections are used to classify communities into transmission categories, which enables the appropriate approach to mass treatment in a community [9]. Existing recommendations on frequency of treatment and target populations (Table 1) were developed with the aim of controlling morbidity associated with schistosomiasis. With the paradigm shift from control to elimination of schistosomiasis, the current recommended treatment strategy and treatment threshold for interventions is not compatible with the permanent interruption of transmission. Furthermore, schistosomiasis is a dynamic disease and prevalence within communities can change rapidly from year to year. Thus contamination of a water body by a few remaining infected individuals can give rise to outbreaks of disease that need to be quickly contained.

**Table 1:** Recommended treatment strategy for schistosomiasis in preventive chemotherapy (WHO, 2006)

Category	Prevalence among school-aged children	Action to be taken	
High-risk community	≥50% by parasitological methods (intestinal and urinary schistosomiasis) Or ≤30% by questionnaire for visible haematuria (urinary schistosomiasis)	Treat all school-age children (enrolled and not enrolled) once a year	Also treat adults considered to be at risk (from special groups to entire communities living in endemic areas; see Annex 6 for details on special groups)
Moderate-risk community	≥10% but <50% by parasitological methods (intestinal and urinary schistosomiasis) Or <30% by questionnaire for visible haematuria (urinary schistosomiasis)	Treat all school-age children (enrolled and not enrolled) once every 2 years	Also treat adults considered to be at risk (special risk groups only; see Annex 6 for details on special groups)
Low-risk community	<10% by parasitological methods (intestinal and urinary schistosomiasis)	Treat all school-age children (enrolled and not enrolled) twice during their primary schooling age (e.g. once on entry and once on exit)	Praziquantel should be available in dispensaries and clinics for treatment of suspected cases

In recent years, the costs of PZQ and the lack of resources were major constraints for the elimination of schistosomiasis. Today, there is a greater impetus, with increasing funding opportunities and donated PZQ by pharmaceutical companies. Time is right to move towards schistosomiasis elimination, and for this challenge there is a need to adapt the current threshold for intervention (i.e. prevalence > 10%) and to define carefully the implementation unit for PZQ mass drug administration. Treatment algorithms should be re-defined based on current knowledge and experiences. WHO has recommended that after achieving morbidity control, preventive chemotherapy should be appropriately adjusted to the new epidemiological conditions by lowering the prevalence risk thresholds. Further, beyond the stage at which elimination as a public-health problem is achieved, a more aggressive strategy will be required in order to attain the more ambitious goal of interrupting transmission [1]. To achieve this goal as set in the WHO Schistosomiasis Strategic Plan 2012–2020, the prevalence of heavy-intensity infections should be reduced to less than 5% in all schistosomiasis-endemic countries by 2020, and to less than 1% by 2025 [65].

### The need for better diagnostic tools

Because of its simplicity and relatively low-cost, the Kato–Katz technique is widely used for epidemiological field surveys and is recommended by the WHO for surveillance and monitoring of schistosomiasis control programmes [66]. Though the specificity is high, the sensitivity of Kato–Katz in single stool sample examination is limited by day-to-day variation in egg excretion rates, thus leading to measurement error in estimating the presence of infection. This is particularly accentuated in areas with high proportions of light intensity infections [67, 68]. In the current era of preventive chemotherapy, the intensification of large-scale interventions and repeated mass deworming will significantly reduce the prevalence and intensities of schistosome infections [69]. As a consequence of the increase of low-intensity infections, less intense infections will be often missed if single stool samples are examined by Kato–Katz method, resulting in significant underestimation of prevalence [67]. Therefore, there is a need to develop and validate more sensitive diagnostic tools for accurate surveillance and monitoring of schistosomiasis control programmes, and for monitoring of drug efficacy. Some studies recommended multiple stool samples in order to avoid underestimating the ‘true’ prevalence and transmission potential of the parasite. Indeed, it was demonstrated that Kato–Katz examination of three instead of one stool specimen increased the sensitivity of helminth diagnosis, most notably for hookworm and schistosomes [70]. However, this has significant cost implications and it is highly time consuming. It is therefore, unlikely that control programmes can easily undertake for multiple sample collections on different days, at more geographical sites.

Several alternative diagnostic tools have been tested for the detection of schistosome infections. The point-of-care urine-based circulating cathodic antigen (POC-CCA) test has been reported as more sensitive than Kato Katz for intestinal schistosomiasis. This test has been widely applied for the diagnosis of *S. mansoni* in Africa [70, 71]. The data from a multi-country study indicated that the POC-CCA assay can contribute greatly to the identification of endemic locations, thereby providing a tool for the more accurate disease mapping needed to properly plan and cost interventions for control, with the ultimate objective of moving toward total elimination [71]. Studies have also demonstrated the higher sensitivity of the circulating anodic antigen (CAA) compared to Kato Katz or urine analysis alone. However, higher prevalence obtained with both CCA and CAA tests argue for the continuation of mass drug administration in endemic zones. The high costs to implement these tests and control interventions may certainly constitute a major constraint. As we move to elimination it may also be appropriate to move away from MDA to a test and treat scenario.

Further efforts should be made to validate other detection tools. The choice of a specific diagnostic assay should be governed by the objective of the activity and according to the status of control [66]. As the accuracy of a given diagnostic technique may vary significantly according to schistosomiasis transmission level, tools should be adapted when moving from morbidity control to elimination of infection. Moving toward the surveillance and elimination phases requires more sensitive techniques such as antibody detection. However, sero-diagnostics tools for detection of schistosome infections require blood sample collection (invasive) and access to affordable, high-quality reagents [72]; all being limiting factors for their integration into large-scale national control programmes. These limitations are amongst the reasons why only a few countries have adopted antibody detection as a key strategy in schistosomiasis diagnosis [73].

As transmission would be the measure of the true end point of elimination, consideration should also be given to the detection of natural schistosome infections in snails and the measurement of the force of infection from cercariae.

### Tackling reservoir hosts

Several species of schistosome are zoonotic and can naturally be transmitted between humans and vertebrate animals. Many domestic and wildlife animals act as reservoir hosts for *S. japonicum* in Asia [74], and the involvement of rodents in the transmission of *S. mansoni* has been demonstrated in Guadeloupe [75]. In Africa, monkeys and baboons are known to be infected by *S. mansoni* in their ecological areas [76]. However, their potential reservoir role in the transmission of the disease and as an impediment to schistosomiasis elimination need to be further investigated. Although *S. haematobium* is assumed human-specific, hybridization within S*. haematobium*-group may constitute a real threat to elimination, and a risk for outbreaks such as in Corsica [77]. The complex population biology and transmission ecology between humans and animal reservoirs affect the success of control programmes, and magnifies the challenges of elimination. Indeed, to eliminate schistosomiasis, one must not only eliminate infection in the human population, but also prevent or eliminate transmission from animal reservoirs [78].

### The need for more funding

In Africa, schistosomiasis control programmes mainly depend on external funds for MDA and often receive donated drugs. If funding ceases, consolidation of achievements made is generally not sustainable, with rapid re-emergence of schistosomiasis as a result. With the intensification of interventions towards schistosomiasis elimination, there is a need to increase funding to support implementation of these interventions. More resources should be mobilized to develop greater multisectoral collaboration in an effort to combat schistosomiasis. The third WHO report on NTDs, “Investing to overcome the global impact of neglected tropical diseases”, recognizes the elimination and control of NTDs as a “litmus test” for universal health coverage; and calls all endemic countries to contribute by increasing their domestic investments to scale-up interventions. NTD control needs to be become an integral part of national health plans and budgets and rely less on foreign aid and charity if it is to achieve universal health coverage [79].

## Conclusion

It is clear that the landscape of schistosomiasis is changing across SSA owing to the many ongoing interventions currently underway. In some regions, country progress may be uneven but in some countries there are real prospects to transition from control into interruption of transmission and ultimately elimination settings. To manage this transition calls for reconsideration of some of the current diagnostic tools and the realignment of existing prevalence treatment thresholds and their interpretation in defining areas where intervention is required. The key challenge will be sustaining and expanding the current donation of praziquantel and judging when it is appropriate to move from MDA to selective treatment, which will ensure that the health system is adapted to respond to these new disease dynamics.

## Additional file

Additional file 1: Multilingual abstracts in the six official working languages of the United Nations. (PDF 442 kb)
