# Supplementary material for: Moving from control to elimination of schistosomiasis in sub-Saharan Africa: time to change and adapt strategies
Source: Infect Dis Poverty. 2017 Feb 20;6:42. doi: 10.1186/s40249-017-0256-8 (PMC5319063; doi:10.1186/s40249-017-0256-8)
Supplement: Additional file 1: — Multilingual abstracts in the six official working languages of the United Nations. (PDF 442 kb) [file 40249_2017_256_MOESM1_ESM.pdf]

Translation of the abstract into the six official working languages of the United Nations

الانتقال من مرحلة السيطرة إلى إبادة مرض الشستوسوما في الدول الإفريقية جنوب الصحراء الكبرى: حان الوقت لتغيير وأقلمة الاستراتيجيات

لويس-ألبيرت تشوم تشينتي، دافيد رولينسون، ج راسل ستاذارد، دافيد مولينو.

ملخص:

مرض الشستوسوما هو مرض ينتقل عبر الماء، ذو أهمية عالمية، وفي أثناء بذل الجهود من أجل السيطرة عليه، فإن خارطة انتشاره تتغير. في قارة أفريقيا جنوب الصحراء الكبرى مثلا، تتغير الخارطة لتصبح أكثر تنوعا، وذلك لوجود أنواع متعددة من الشستوسوما والتي تستجيب بطرق مختلفة للعلاجات الوقائية المستخدمة والإجراءات الوقائية التي تنفذ في القطاعات المختلفة. وأهم ما ترتكز عليه عملية الوقاية هو إعطاء عقار برازيكوانتل عن طريق البرامج العلاجية الشاملة للأشخاص الذين يكونون، أو يشك في كونهم معرضون للعدوى والمرض. في بعض الدول، قد يكون التقدم المحرز مختلف في أقاليمها المختلفة، ولكن في بعض المواقع المحددة هناك امكانيات حقيقية للتحول من مرحلة السيطرة على العدوى إلى عرقلة الانتقال، وصولا للإبادة في النهاية. وإدارة هذا التحول بحاجة لإعادة نظر في بعض الأدوات التشخيصية المستخدمة حاليا في عمليات المسح بالإضافة إلى تقليل عتبة معدل تواجد العدوى المستخدمة لإطلاق عمليات العلاج الشامل. ومن التحديات الأساسية القادمة المحافظة على كمية البرازيكوانتل المتبرع بها وتوسعتها إن أمكن لتشمل الجماعات التي لم تشمل قبلا، ثم تقرير الوقت المناسب للانتقال من عملية العلاج الشامل إلى المعالجة الانتقائية للحالات. وبتنفيذ تلك الخطوات نضمن كون النظام الصحي، محدّثا، ومعدّا واقتصاديا من أجل الاستجابة لهذه الأوضاع المتغيرة أثناء مثابرتنا للأمام تجاه هدف 2020 وما بعده.

Translated from English version into Arabic by SAlkhodair, through

TRANSLATORS
WITHOUT BORDERS

撒哈拉以南非洲血吸虫病从控制到消除阶段的策略转变与适应

Louis-Albert Tchuem Tchuenté, David Rollinson, J. Russell Stothard, David Molyneux

摘要

血吸虫病是一种具有全球影响的水源性寄生虫病，随着控制措施的持续推行，其疾病流行景观也一直在变化。例如，在撒哈拉以南的非洲地区，由于几种血吸虫虫种以不同的方式对正在进行的预防性化疗和跨领域干预作出反应，其景观越来越异质化。预防性化疗主要通过大规模药物治疗将吡喹酮递送至显示/或假定有感染风险的地区和流行区。在一些国家，尽管区域进展可能不平衡，但某些地区非常有可能实现从控制过渡到阻断直至消除。如要把握这种转变，需要重新考虑一些目前部署在监测系统的诊断工具，并下调现有流行率阈值以开启大规模治疗。如果可能，把目前捐赠的吡喹酮扩大至被忽视的群体将成为突破性进展，然后在适当时间从大规模药物供给转向选择性治疗。如此一来，将确保改善医疗体系，尽快显示成本效益，以应对这些不断变化的疾病动态，迈向并超越 2020 年目标。

Translated from English version into Chinese by Jin Chen, edited by Pin Yang

CDC CHINESE CENTER FOR DISEASE CONTROL AND PREVENTION
NATIONAL INSTITUTE OF PARASITIC DISEASES

De la lutte à l’élimination de la schistosomiase en Afrique subsaharienne : le moment est venu de changer et d’adapter les stratégies

Louis-Albert Tchuem Tchuenté, David Rollinson, J. Russell Stothard et David Molyneux

Résumé

La schistosomiase est une maladie parasitaire hydrique d'importance mondiale dont le contexte endémique est en train d'évoluer. Par exemple, en Afrique subsaharienne, le contexte devient de plus en plus hétérogène du fait de l'existence de plusieurs espèces de *Schistosoma* qui réagissent de différentes manières à la chimiothérapie préventive et aux interventions intersectorielles actuellement en vigueur. La chimiothérapie préventive repose principalement sur l'administration massive du praziquantel à ceux qui sont, ou sont présumés être, à risque de contracter la maladie. Dans certains pays, on observe des disparités régionales en matière de progrès, mais dans certains endroits, il existe des perspectives réelles de passer de la lutte à l'interruption de la transmission, voire de l'élimination de la maladie. La gestion de cette phase de transition passe par le réexamen de certains outils de diagnostic utilisés actuellement pour la surveillance ainsi que par le réajustement à la baisse de certains seuils de prévalence existants pour permettre un traitement de masse. L'un des principaux défis sera de poursuivre le traitement au praziquantel et, si possible, de l'étendre aux groupes actuellement négligés, pour ensuite, au besoin, passer de l'administration massive de médicaments au traitement sélectif. De cette façon, on garantit un système de santé adapté, prêt et rentable pour affronter la nouvelle dynamique de la maladie à mesure que nous progressons vers les objectifs de 2020 et au-delà.

Translated from English version into French by Sabiha Azibi, through

Переход от борьбы с шистосомозом к его ликвидации в Чёрной Африке: время менять и приспосабливать стратегии

Луи-Альберт Чуем Чуэнте (Louis-Albert Tchuem Tchuenté), Дэвид Роллинсон (David Rollinson), Дж. Расселл Стофард (J. Russell Stothard), Дэвид Молино (David Molyneux)

Аннотация

Шистосомоз – это передающееся через воду паразитарное заболевание глобального значения. Непрекращающаяся борьба с этой болезнью приводит к изменению её эндемической картины. Например, в Чёрной Африке она становится ещё более гетерогенной, так как существует несколько видов *шистосом,* на которые продолжительная профилактическая химиотерапия оказывает различное воздействие. Поэтому в настоящее время применяются методы межсекторального вмешательства. Главным направлением профилактической химиотерапии является массовое назначение празиквантела тем, кто подвергается или может быть подвержен инфицированию и заболеванию. В некоторых странах прогресс в регионах может быть неравномерным, однако в определённых местностях вполне реален переход от борьбы с болезнью к прекращению передачи возбудителя и в перспективе полной ликвидации. Управление таким переходом требует пересмотра отдельных используемых сейчас диагностических средств наблюдения и снижения существующих пороговых величин заболеваемости, которые приводят к массовому лечению. Основная трудность будет заключаться в сохранении и по возможности увеличении количества выдаваемого сейчас бесплатно празиквантела группам, которые в настоящее время игнорируются, затем в определении момента, когда следует перейти от массового лечения к выборочному. Тем самым система здравоохранения будет приспособлена, подготовлена и рентабельна для того, чтобы реагировать на меняющуюся динамику болезни, в то время как мы работаем над достижением целей 2020 года и последующих лет.

Translated from English version into Russian by Natalia Potashnik, through

Del control a la erradicación de la esquistosomiasis en el África subsahariana: modificación y adaptación de las estrategias

Autores: Louis-Albert Tchuem Tchuenté, David Rollinson, J. Russell Stothard, David Molyneux

Resumen

La perspectiva endémica de la esquistosomiasis, una enfermedad parasitaria extendida por todo el mundo que se transmite a través del agua, está cambiando debido al control continuado de la enfermedad. Por ejemplo; en el África subsahariana cada vez es más heterogénea, ya que hay varias especies de esquistosoma que responden de distinta forma a la quimioterapia preventiva continua y a las intervenciones intersectoriales que se llevan a cabo en la actualidad. La quimioterapia preventiva continuada se centra principalmente en la administración masiva de praziquantel a quienes presentan o pueden presentar riesgo de infección y de contraer la enfermedad. Pese a que en algunos países el progreso de una región a otra puede ser desigual, en ciertas zonas las expectativas de transición del mero control de la enfermedad a la interrupción de su transmisión y, en última instancia, a su erradicación, son muy reales. Dicha transición requiere reconsiderar ciertas herramientas de diagnóstico que se utilizan en la actualidad para monitorizar la enfermedad y una reducción de los actuales umbrales de prevalencia a fin de facilitar el tratamiento masivo. Uno de los retos clave será mantener y, si es posible, ampliar la administración actual de praziquantel a grupos que a día de hoy no son objetivo, para luego determinar cuándo procede pasar de dicha administración masiva a un tratamiento selectivo. De este modo, el sistema sanitario estará adaptado y optimizado (también en lo relativo a los costes) para responder a estas dinámicas de cambio de la enfermedad según avanzamos hacia los objetivos para 2020 y años sucesivos.

Translated from English version into Spanish by Marta Callava Linares, through

TRANSLATORS
WITHOUT BORDERS
